# Comparative effects of high pressure processing and heat treatment on in vitro digestibility of pea protein and starch

**DOI:** 10.1038/s41538-021-00116-0

**Published:** 2022-01-12

**Authors:** Alexandra E. Hall, Carmen I. Moraru

**Affiliations:** Department of Food Science, Cornell University, Ithaca, NY 14853 USA

**Keywords:** Chemistry, Agriculture

## Abstract

The effects of high-pressure processing (HPP) and heat treatment on the digestibility of protein and starch in pea protein concentrate (PPC) were investigated. Samples of PPC with 5% (5 P) and 15% (15 P) protein were treated by HPP (600 MPa/5 °C/4 min) or heat (95 °C/15 min) and their in vitro static and dynamic digestibility were compared to untreated controls. HPP-treated PPC underwent a greater degree of proteolysis and showed different peptide patterns after static gastric digestion compared to untreated and heat-treated PPC. Differences in protein digestibility among treatments during dynamic digestion were only significant (*p* < 0.05) during the first 20 min of jejunal, ileal, and total digestion for 5 P, and during the first 60 min of ileal digestion for 15 P. Neither static nor dynamic starch digestibility were dependent on treatment. HPP did not reduce trypsin inhibitor activity, whereas heat treatment reduced it by ~70%. HPP-induced structural modifications of proteins and starch did not affect their overall in vitro digestibility but enhanced gastric proteolysis.

## Introduction

High-pressure processing (HPP) is a nonthermal food processing technology that can be used as an alternative to thermal pasteurization, without the detrimental sensory and nutritional changes to the food matrix induced by heat^1^. The pressures (200–600 MPa) used in typical HPP treatments do not break covalent bonds, but rather shift molecular structures towards reduced volume states, resulting in the stabilization of hydrogen bonds and the disruption of hydrophobic and electrostatic interactions^2^. Consequently, HPP can induce structural and functional modification of food polymers, which can be harnessed to achieve food products with unique textures and high quality^1,3^. Our group recently showed that pressure treatments induce structural modifications in milk and pea proteins, and a range of structures and textures can be created by controlling the pressure level and protein concentration^4–6^. These pressure-induced structural modifications of food biopolymers can also alter the accessibility of enzymatic cleavage sites, and thus their susceptibility for enzymatic hydrolysis^7^, which has implications for both the shelf life^8^ and digestibility of pressure-treated products.

Exploring the HPP treatment of pulses (peas, chickpeas, lentils, and beans) is very compelling since the utilization of pulses as a plant protein source has garnered considerable interest in recent years. New food processing technologies like HPP afford new, untapped opportunities for using pulse proteins to create new food products. However, a known challenge of pulse proteins compared to animal-based proteins is their lower protein quality. Protein quality is dependent on the amino acid profile and digestibility of the protein, as well as the bioavailability of amino acids^9^. In addition to having limited amino acid profiles, pulses have low protein digestibility due to specific molecular conformations that impede accessibility of enzymatic cleavage sites, and antinutritional factors like trypsin inhibitors that inhibit protein digestion^7,10^. Understanding the impact of HPP on the digestibility and trypsin inhibitor activity of pulse proteins is very important, as this can elucidate the effect of this technology on protein quality. Previous studies on HPP-treated pulse or soy protein have reported varying trends in digestibility, however, many of these studies were conducted at relatively low protein concentration, or did not include all the major digestive enzymes^11–20^.

Commercially available pea protein preparations also contain a significant amount of starch. It has been reported that pressure-treated starch retains a greater degree of granule structure compared to heat treatment, and thus it may be more resistant to digestion than heat-treated starch^4,21^. This is not necessarily an undesirable effect, since resistant starch was linked to numerous health benefits such as lowering insulin and glycemic responses, and improving gut microflora health^22^. The effect of HPP on starch digestibility is also of interest.

Therefore, the objective of this study was to evaluate the effects of HPP on the in vitro digestibility of proteins and starch, as well as trypsin inhibitor activity, in comparison with those of heat treatment. Commercially available pea protein concentrate (PPC), a complex system containing primarily globulin (legumin, vicilin, and convicilin) and albumin proteins^23^ was used as a source of pea protein and pea starch. Two pea protein concentrations were used to emulate protein concentrations characteristic of food applications: a low concentration (5% w/w; 5 P), relevant to protein-fortified beverages, and a high concentration (15% w/w; 15 P), conducive toward gel formation^5^. HPP parameters commonly used in the food industry for microbial inactivation, which were also proven to induce protein and starch structural transformations^4,24^, were used (600 MPa/5 °C/4 min). Heat treatments at 95 °C/15 min were used for comparison, as these conditions were found to induce pea protein gel network formation at high protein concentrations^5^. While a few studies have examined the digestibility of thermally-induced plant protein gel networks^25,26^, no data has been published to date on the digestibility of pressure-induced plant protein gel networks. This study will inform food-related applications of HPP-treated pea protein ingredients.

## Results

After both HPP and heat treatment, the 5 P samples remained as concentrated protein solutions, while the 15 P samples formed self-standing gel structures. Detailed information regarding the structure and rheological properties of these protein systems can be found in the studies by Sim et al.^4,5^.

### Assessment of proteins and peptide fractions in in vitro PPC digesta using SDS-PAGE

**Fig. 1:**
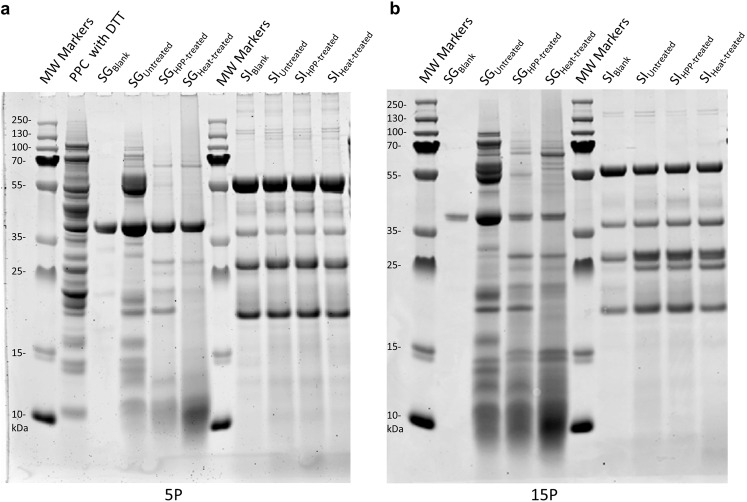
SDS-PAGE of unfiltered static model digesta. Representative gels of **a** untreated undigested pea protein concentrate (PPC) and unfiltered SG and SI after static in vitro digestion of PPC with 5% (w/w) protein (5 P) and **b** unfiltered SG and SI after static in vitro digestion of PPC with 15% (w/w) protein (15 P). SG was the static model cumulative gastric digesta and SI was the static model cumulative intestinal digesta, see Table 1 for all symbol descriptions. Gels were run for each independent replicate of digestions in technical duplicate. DTT was added to the untreated undigested PPC to distinguish subunits. Molecular weight markers were included for reference. The major protein bands for PPC were at 71 kDa for convicilin subunits; 50, 30–35, and 13–20 kDa for vicilin subunits; 40 and 20 kDa for legumin subunits; and 25–26, 4, and 6 kDa for albumin polypeptides^29^. Blank digestions consisting of water in replace of PPC were conducted to discriminate enzymes from PPC. Pepsin was localized around 35 kDa on the gels, whereas pancreatic lipases, amylases, and proteases were around 20–25 and 40–55 kDa^30^.

Untreated (control), HPP-treated, and heat-treated 5 P and 15 P were subjected to static in vitro digestion, and digesta were analyzed by sodium dodecyl sulfate-polyacrylamide gel electrophoresis (SDS-PAGE) to determine treatment-dependent changes in hydrolyzed protein band patterns, and the concentration of dye-binding proteins and peptides. The dye-binding proteins and peptides present in the SDS-PAGE gels in Fig. 1a, b represent intact proteins and peptides larger than 3 kDa, since Coomassie blue dye-binding is usually limited to proteins and peptides greater than 3 kDa^27^. However, since dye-binding is also dependent on the presence of basic or aromatic residues^28^, a precise molecular weight cutoff for the different bands could not be established. Therefore, to avoid inaccuracies, the quantitative evaluation of the concentration of dye-binding proteins and peptides found in the digesta was performed in terms of relative concentrations between treated vs untreated samples and will be referred to as “relative protein concentration”. A value of the relative protein concentration < 1 indicates that treatment increased the digestibility of the protein sample compared to the untreated one.

Many of the protein and peptide fractions found in the untreated undigested PPC samples were present in all static gastric digesta (SG) of 5 P and 15 P samples (Fig. 1a, b), which indicates that they were fairly resistant to extensive proteolysis in the gastric phase. SDS-PAGE gels for the untreated undigested PPC showed a complex pattern (Fig. 1a), with major protein bands located at 71 kDa (convicilin subunits); 50, 30–35, and 13–20 kDa (vicilin subunits); 40 and 20 kDa (legumin subunits); and 25–26, 6, and 4 kDa (albumin polypeptides)^29^. For each treatment type, the band patterns indicating the progression of enzymatic hydrolysis during gastric digestion for 5 P and 15 P were consistent across replicates. Band patterns were however different between treatments, suggesting that the dynamics of pepsin cleavage of PPC was dependent on treatment type. For instance, untreated 5 P and 15 P SG had considerably greater peptide and protein content around 55–70 kDa compared to HPP-treated and heat-treated 5 P and 15 P SG, and heat-treated 5 P and 15 P SG had considerably greater peptide content around 10 kDa compared to the untreated and HPP-treated 5 P and 15 P SG.

**Fig. 2:**
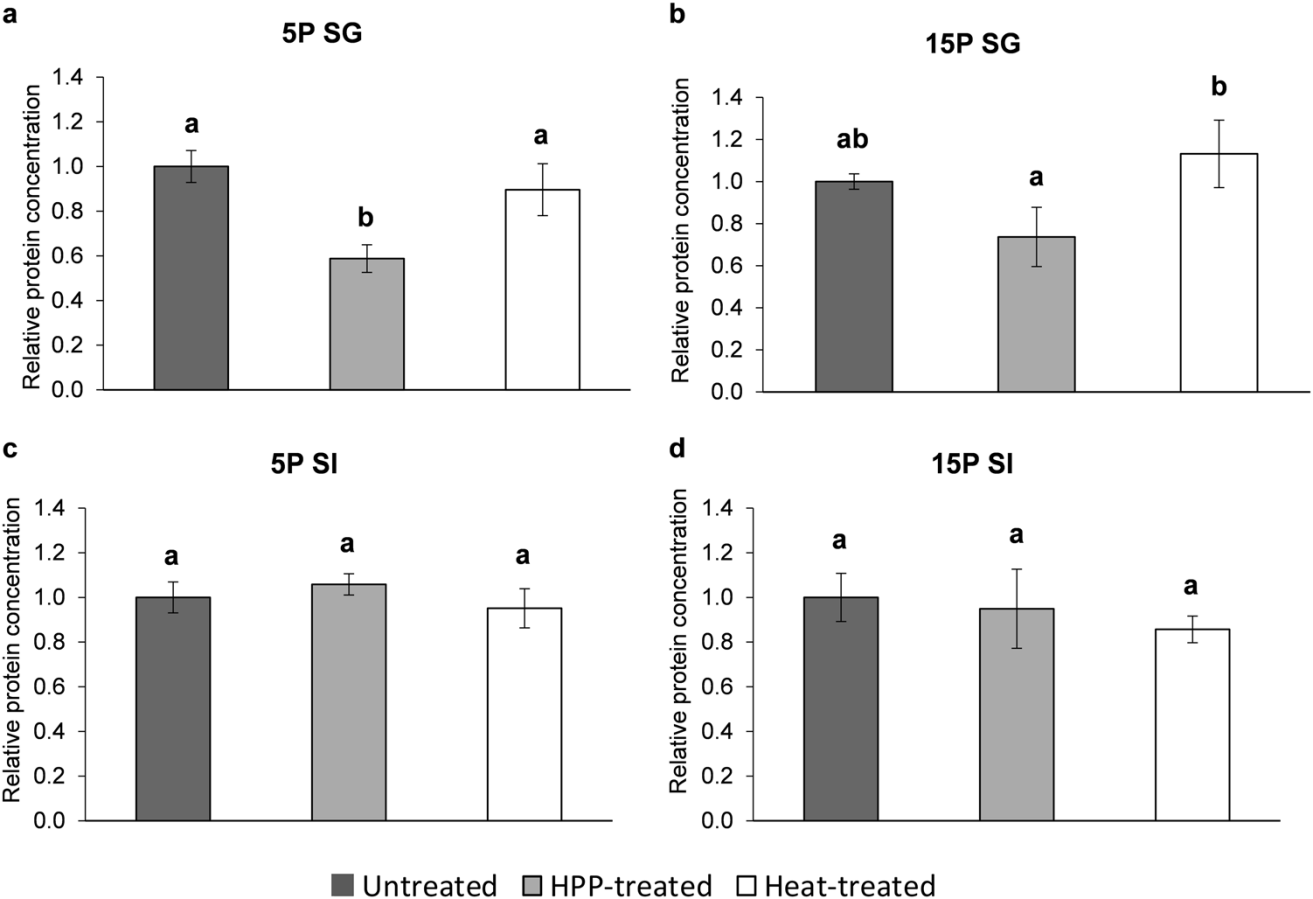
Average relative protein concentration in unfiltered SG and SI separated by SDS-PAGE. The average relative protein concentrations (unitless) in unfiltered SG after static gastric in vitro digestion of **a** pea protein concentrate with 5% (w/w) protein (5 P) and **b** pea protein concentrate with 15% (w/w) protein (15 P), and in unfiltered SI after completion of static in vitro digestion of **c** 5 P and **d** 15 P were calculated with respect to the average untreated protein concentration for SG and SI of 5 P and 15 P, separately (average protein concentration/average protein concentration of untreated). SG was the static model cumulative gastric digesta and SI was the static model cumulative intestinal digesta, see Table 1 for all symbol descriptions. Gels were run for each independent replicate of digestions in technical duplicate, where the protein concentrations for each independent digestion were an average of the technical duplicates. Bar height represents the average relative protein concentration for the three independent digestions. Error bars display the standard deviation. Letters above the bars indicate statistically significant differences (*p* < 0.05). SG and SI were analyzed by the statistical model separately.

The relative protein concentration in the SG was the lowest for the HPP-treated PPC (Fig. 2a, b): HPP-treated 5 P SG had a relative protein concentration of 0.59 ± 0.06, compared to 0.90 ± 0.12 for heat-treated 5 P SG and 1.00 ± 0.07 for untreated 5 P SG. The differences in gastric digestibility between the untreated and HPP-treated 5 P, and between the HPP-treated and heat-treated 5 P and 15 P were statistically significant (*p* < 0.05). The gastric digestibility of the HPP-treated 15 P was not significantly different than the untreated 15 P (*p* > 0.05).

The proteins detected in all static intestinal (SI) digesta, for both 5 P and 15 P, were identified as digestive enzymes. In Fig. 1a, b, the SG_Blank_ and SI_Blank_ wells represent the digestive enzymes present in the gastric and intestinal digesta, respectively. The pancreatic lipases, amylases, and proteases were present in the intestinal wells at 20–25 and 40–55 kDa, while pepsin was located around 35 kDa^30^. This suggests that most PPC proteins were hydrolyzed into small peptides (~<3 kDa) and free amino acids by the end of the intestinal phase. There were no significant differences (*p* > 0.05) in relative protein concentrations across treatment types in SI of 5 P and 15 P (Fig. 2c, d). The raw data for the SDS-PAGE quantitative analysis is shown in Supplementary Table 1a, b.

### Assessment of peptides < 10 kDa in the in vitro PPC digesta

The bicinchoninic acid (BCA) assay was used to determine the concentration of peptides (roughly tripeptides—10 kDa) released during in vitro static and dynamic digestion for all treatments, and the differences among treatments.

**Fig. 3:**
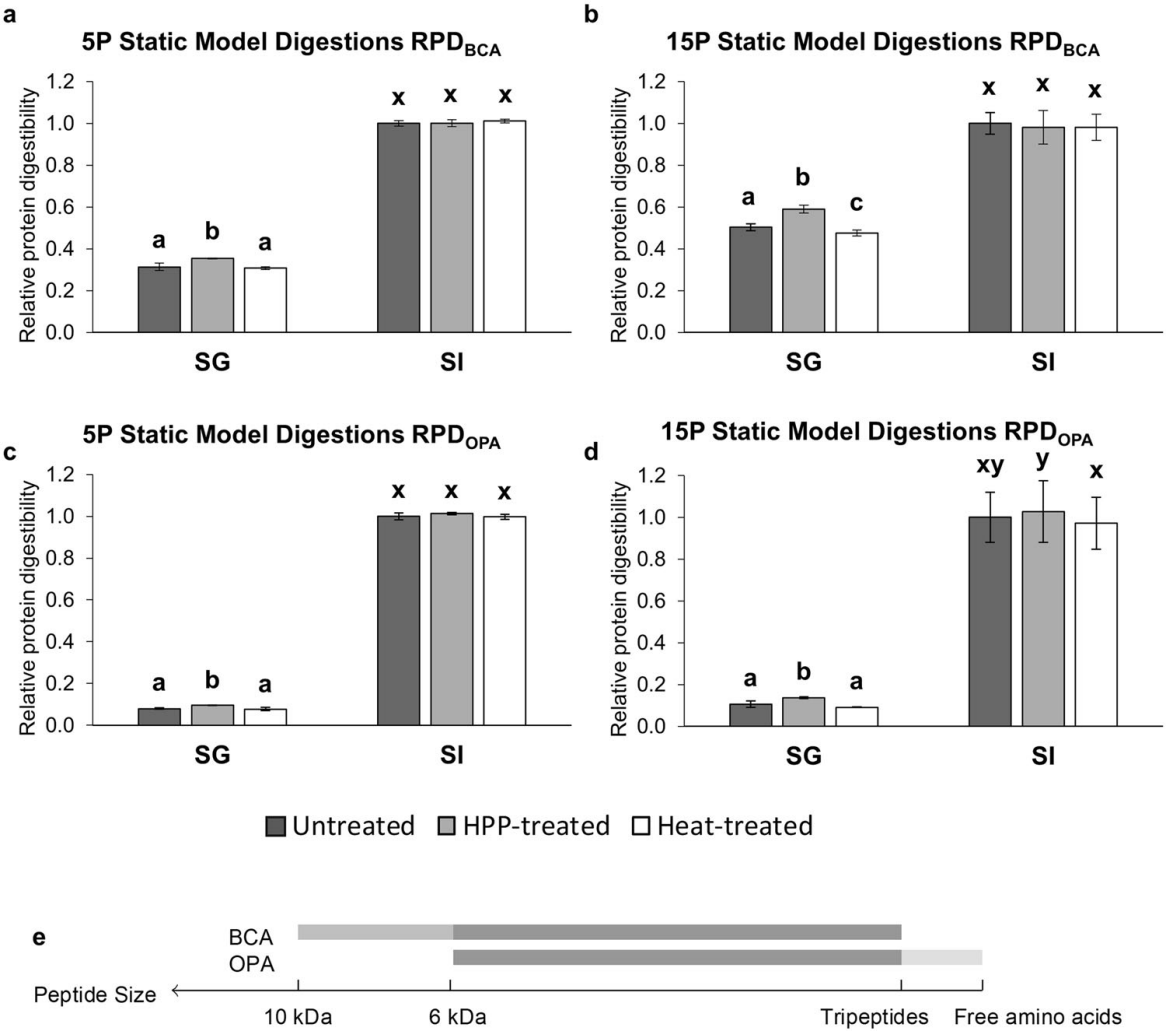
Average relative protein digestibility as determined by the BCA (RPD_BCA_) and OPA (RPD_OPA_) assays for static digestions of pea protein concentrate with 5% (w/w) protein (5 P) and 15% (w/w) protein (15 P). The average relative protein digestibility (unitless) for **a** 5 P RPD_BCA_, **b** 15 P RPD_BCA_, **c** 5 P RPD_OPA_, and **d** 15 P RPD_OPA_ was calculated with respect to the average SI_Untreated_ protein digestibility for each assay at each protein concentration (average % digested protein/average % digested protein of SI_Untreated_). SG was the static model cumulative gastric digesta and SI was the static model cumulative intestinal digesta, see Table 1 for all symbol descriptions. The BCA and OPA assays were performed on each independent replicate of digestions in technical triplicate, where the % digested protein for each independent digestion was an average of the technical triplicates. Bar heights represent the average RPD_BCA_ and RPD_OPA_ for the three independent digestions. Error bars display the standard deviation. Letters above the bars indicate statistically significant differences (*p* < 0.05). SG and SI were analyzed by the statistical model separately. **e** A generalized overview of protein hydrolysates detected by the BCA vs. OPA assays. The dark gray region indicates the area of overlap for peptides detected by both assays. The molecular weight cutoffs are approximations. The 10 kDa cutoff for the BCA assay is due to the molecular weight cutoff for the ultrafiltration step.

For the static digestion, at the end of the gastric phase, relative protein digestibility by the BCA assay (RPD_BCA_), for both 5 P and 15 P, followed the trend: HPP-treated > untreated > heat-treated (Fig. 3a and b). The RPD_BCA_ of HPP-treated PPC after the gastric phase was significantly greater (*p* < 0.05) than that of the untreated and heat-treated PPC, for both 5 P and 15 P, suggesting that pressure-induced changes of the pea proteins enhanced the extent of pepsin proteolysis during gastric static digestion. Significant differences in gastric RPD_BCA_ between untreated and heat-treated PPC were found for 15 P (*p* < 0.05) but not for 5 P (*p* > 0.05), suggesting that heat treatments of the higher pea protein concentration limited the gastric proteolytic enzyme accessibility to the proteins. At the end of the intestinal phase of the static digestion, overall RPD_BCA_ were comparable for all samples and all treatments. Supplementary Table 1a, b show the BCA assay raw data.

**Fig. 4:**
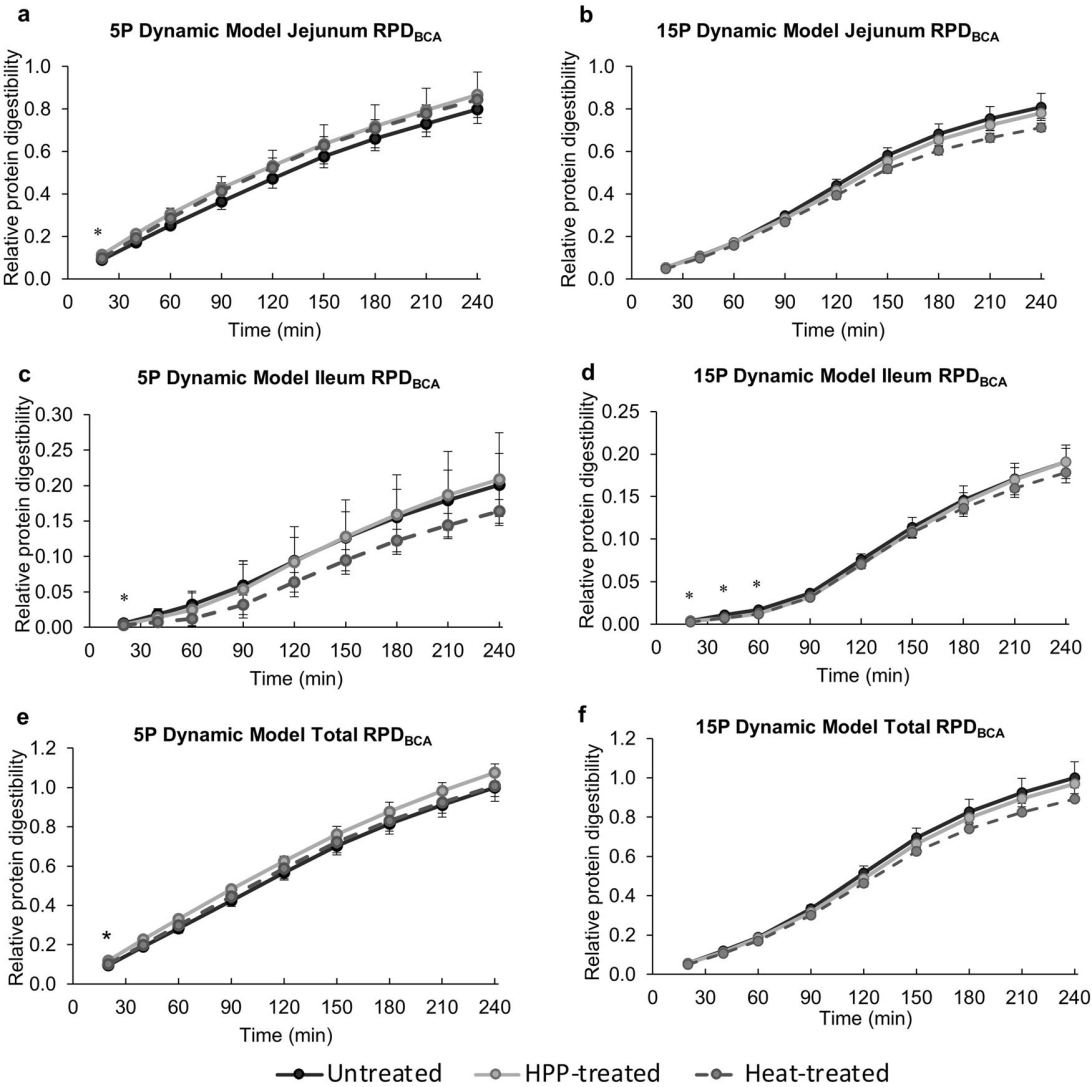
Average cumulative relative protein digestibility as determined by the BCA assay (RPD_BCA_) over time for dynamic digestions of pea protein concentrate with 5% (w/w) protein (5 P) and 15% (w/w) protein (15 P). The average cumulative RPD_BCA_ (unitless) for **a** 5 P and **b** 15 P digesta of the jejunum, **c** 5 P and **d** 15 P digesta of the ileum, **e** 5 P and **f** 15 P total (jejunum + ileum) digesta was calculated with respect to the average cumulative RPD_BCA_ of DCT_240,Untreated_ for the two concentrations separately (average % digested protein/average % digested protein of DCT_240,Untreated_). DCT_240,Untreated_ is the dynamic model cumulative total (jejunal + ileal) digesta for untreated samples after the full 240 min digestion, see Table 1 for all symbol descriptions. The BCA assay was performed on each independent replicate of digestions in technical duplicate, where the % digested protein for each independent digestion was an average of the technical duplicates. Points represent the average cumulative RPD_BCA_ for the three independent digestions. Error bars display the standard deviation. Statistical analysis of effect of treatment type on digestibility was performed at each time point of the jejunum, ileum, and total (jejunum + ileum) digestion, and asterisks indicate the presence of statistically significant pairwise comparisons (*p* < 0.05).

In the dynamic digestion, the 5 P ileal and 15 P jejunal, ileal, and total cumulative RPD_BCA_ followed a sigmoidal curve over time (Fig. 4b–d, f), indicating an initial lag phase, whereas the 5 P jejunal and total cumulative RPD_BCA_ did not show an initial slower rate of digestion (Fig. 4a, e). Cumulative jejunal and total RPD_BCA_ followed the general trend: HPP-treated > heat-treated > untreated for 5 P, and untreated > HPP-treated > heat-treated for 15 P. These differences may be due to the structural differences between the 5 P and 15 P samples treated by either pressure or heat treatment. Pressure-induced gels are predominantly held by weak physical bonds, while heat-formed gels are predominantly bound by covalent S-S bonds^5,6,25^, which could render the latter less digestible. After ileal digestion, heat-treated PPC had generally lower cumulative RPD_BCA_ than untreated and HPP-treated PPC, but these differences were not statistically significant. The only statistically significant differences in RPD_BCA_ between treatments occurred during the first 20 min of jejunal, ileal, and total digestion for 5 P, and the first 60 min of ileal digestion for 15 P. For 5 P: DCJ_20,HPP-treated_ > DCJ_20,Untreated_, DCI_20,Untreated_ > DCI_20,Heat-treated_, DCT_20,HPP-treated_ > DCT_20,Untreated_; for 15 P: DCI_20,Untreated_ > DCI_20,Heat-treated_, DCI_20,HPP-treated_ > DCI_20,Heat-treated_, DCI_40,Untreated_ > DCI_40,HPP-treated_, DCI_40,Untreated_ > DCI_40,Heat-treated_, DCI_60,Untreated_ > DCI_60,HPP-treated_, and DCI_60,Untreated_ > DCI_60,Heat-treated_ (p < 0.05); where DCJ, DCI, and DCT refer to dynamic cumulative jejunal, ileal, and total digesta, respectively (Table 1). Hence, during dynamic digestion, treatment type had a significant effect only on RPD_BCA_ during the initial time points of digestion. Supplementary Table 2a, b include the complete BCA assay raw data.

**Table 1:** Description of sample, digestion, and analyses symbols.

Sample, digestion, and analyses symbols	
Symbol	Description
HPP	High pressure processing
PPC	Pea protein concentrate
5 P	Pea protein concentrate samples with 5% (w/w) protein
15 P	Pea protein concentrate samples with 15% (w/w) protein and 1.19% (w/w) starch
SSF	Simulated salivary fluid
SGF	Simulated gastric fluid
SIF	Simulated intestinal fluid
TIM-1	TNO gastro-intestinal model 1
SDS-PAGE	Sodium dodecyl sulfate-polyacrylamide gel electrophoresis
BCA	Protein assay to determine % protein hydrolyzed to peptides < 10 kDa but > dipeptides
OPA	Protein assay to determine % protein hydrolyzed to peptides < 6 kDa and free amino acids
RPD_BCA_	Relative protein digestibility as determined by the BCA assay
RPD_OPA_	Relative protein digestibility as determined by the OPA assay
RSD	Relative starch digestibility
SG	Static model cumulative gastric digesta
SI	Static model cumulative intestinal digesta
DCJ	Dynamic model cumulative jejunal digesta
DCI	Dynamic model cumulative ileal digesta
DCT	Dynamic model cumulative total (jejunal + ileal) digesta

*Note*: Subscripts designate time points and treatment-type for dynamic cumulative digesta.

### Assessment of peptides < 6 kDa and amino acids in the in vitro PPC digesta

The *o*-phthaldialdehyde (OPA) assay was used to determine changes in the concentration of small peptides (roughly < 6 kDa) and free amino acids released during in vitro static digestion. For the static gastric and intestinal digestion of 5 P and 15 P, the general trend in relative protein digestibility by the OPA assay (RPD_OPA_) was: HPP-treated > untreated > heat-treated (Fig. 3c, d). HPP-treated PPC had significantly greater gastric RPD_OPA_ than untreated and heat-treated PPC, for both 5 P and 15 P (*p* < 0.05). Hence, HPP-treated PPC was more susceptible to hydrolysis to small peptides and free amino acids compared to untreated and heat-treated PPC during static gastric digestion. At the end of the intestinal phase of static digestion, the only statistically significant difference in RPD_OPA_ was between HPP-treated and heat-treated 15 P (*p* < 0.05). HPP-treated 15 P may have undergone more extensive hydrolysis to small peptides and free amino acids throughout static digestion compared to heat treatment, likely due to the physical nature of the pressure-treated gels as compared to the covalently bonded gels, as discussed before. Supplementary Table 1a, b contains the OPA assay raw data.

### Effect of HPP and heat treatment on relative trypsin inhibitor activity

**Fig. 5:**
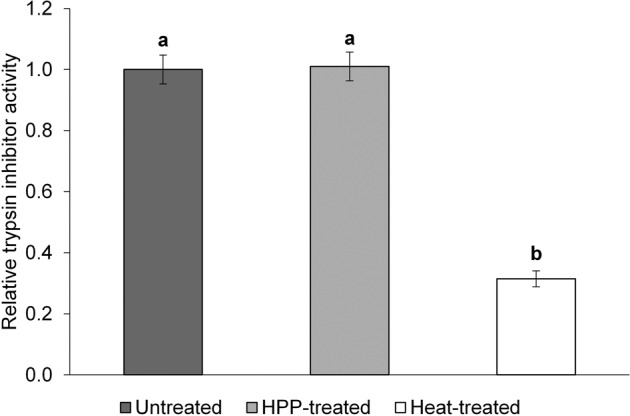
Average relative trypsin inhibitor activity. The trypsin inhibitor activity assay was performed independently in triplicate, where the trypsin inhibitor activity (mg trypsin inhibited/g protein) for each independent replicate was an average of three technical replicates. Bars represent the average relative trypsin inhibitor activity (unitless) with respect to the average untreated trypsin inhibitor activity (average trypsin inhibitor activity/average trypsin inhibitor activity of untreated). Error bars display the standard deviation. Letters above the bars indicate statistically significant differences (*p* < 0.05).

The trypsin inhibitor activity was determined only for the 5 P samples. The relative trypsin inhibitor activity of the heat-treated 5 P (0.31 ± 0.03) was nearly 70% lower than that of the untreated (1.00 ± 0.05) and HPP-treated (1.01 ± 0.05) samples (Fig. 5). Differences in relative trypsin inhibitor activity between the heat-treated 5 P vs. the untreated and HPP-treated 5 P were significantly different (*p* < 0.05). This indicates that HPP treatment was not effective at inactivating trypsin inhibitors in 5 P, whereas the heat treatment was effective. Supplementary Table 3 includes trypsin inhibitor activity raw data.

### Effect of HPP and heat treatment on pea starch digestibility

Since PPC also contained a significant amount (4.3% dry weight) of starch, in vitro starch digestibility after the different treatments were also assessed. This was conducted only for the 15 P samples, due to the low starch concentration for the 5 P samples. The concentration of hydrolyzed glucose monomers released upon static and dynamic in vitro digestion was measured and used to determine relative starch digestibility (RSD).

After static digestion, RSD for untreated (1.00 ± 0.04), HPP-treated (1.01 ± 0.01), and heat-treated (0.99 ± 0.02) samples were not statistically different from each other (*p* > 0.05), indicating that neither HPP nor the heat treatment affected overall starch digestibility by the conclusion of static digestion. Supplementary Table 1b contains the glucose assay raw data.

**Fig. 6:**
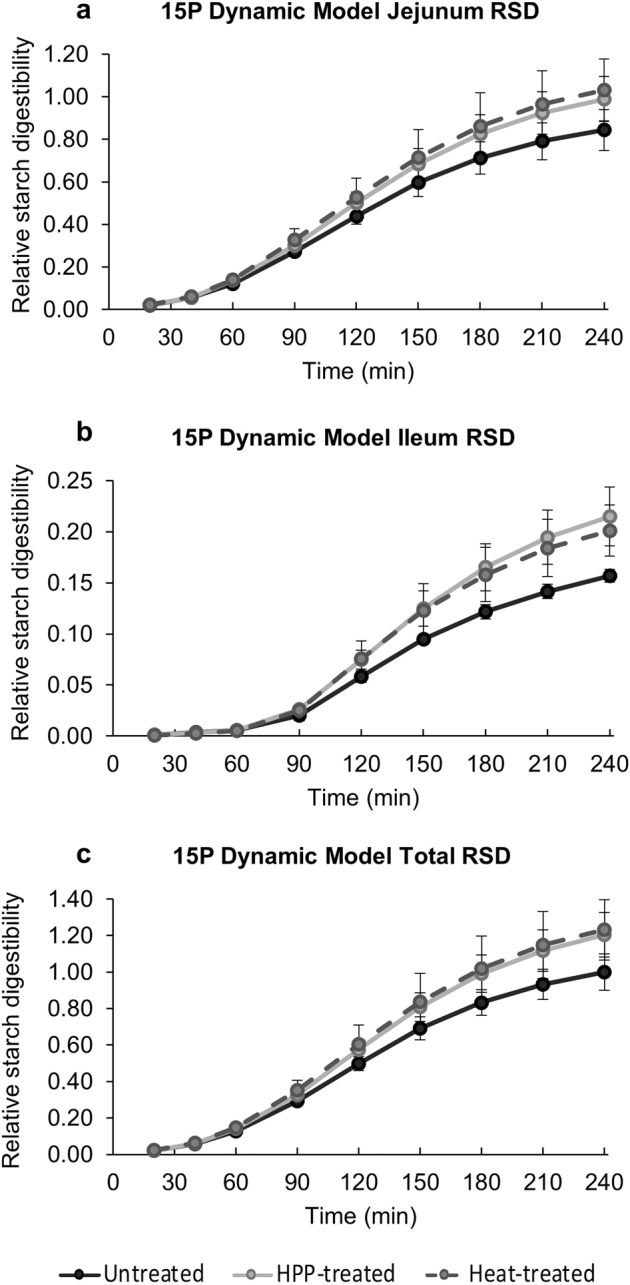
Average cumulative relative starch digestibility (RSD) over time for dynamic digestions of pea protein concentrate with 15% (w/w) protein (15 P). The average cumulative RSD (unitless) for **a** 15 P digesta of the jejunum, **b** 15 P digesta of the ileum, and **c** 15 P total (jejunum + ileum) digesta was calculated with respect to the average cumulative RSD of 15 P DCT_240,Untreated_ (average % digested starch/average % digested starch of DCT_240,Untreated_). DCT_240,Untreated_ is the dynamic model cumulative total (jejunal + ileal) digesta for untreated samples after the full 240 min digestion, see Table 1 for all symbol descriptions. The glucose assay was performed on each independent replicate of digestions in technical duplicate, where the % digested starch for each independent digestion was an average of the technical duplicates. Points represent the average cumulative RSD for the three independent digestions. Error bars display the standard deviation. Statistical analysis of the effect of treatment type on digestibility was performed at each time point of the jejunum, ileum, and total (jejunum + ileum) digestion. No statistical comparisons were significant (*p* > 0.05).

**Fig. 7:**
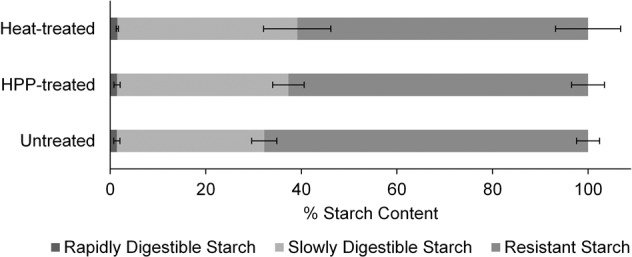
Average percent rapidly digestible, slowly digestible, and resistant starch content based on dynamic digestions of pea protein concentrate with 15% (w/w) protein (15 P). The percent of glucose released from starch during the first 20 and 20–120 min of dynamic digestion was categorized as rapidly digestible and slowly digestible starch, respectively. The remaining starch that did not break down to glucose after 120 min of dynamic digestion was deemed resistant starch^68^. The glucose assay was performed on each independent replicate of digestions in technical duplicate, where the percent starch content for each independent digestion was an average of the technical duplicates. Bars represent the average percent starch content for the three independent digestions. Error bars display the standard deviation. Statistical comparisons of starch content by treatment type were not significant (*p* < 0.05).

During dynamic digestion, the jejunal, ileal, and total cumulative RSD followed a sigmoidal curve overtime for all samples (Fig. 6a–c). Cumulative jejunal and total RSD followed the general trend: heat-treated > HPP-treated > untreated, although no differences were statistically significant at any time point (p > 0.05). Cumulative ileal RSD followed the general trend: HPP-treated > heat-treated > untreated, although none of the differences observed were statistically significant at any time point as well. Untreated 15 P had the greatest resistant starch content and the lowest slowly digestible and rapidly digestible starch content, whereas heat-treated 15 P had the lowest resistant starch content and the greatest slowly digestible and rapidly digestible starch content (Fig. 7). HPP-treated 15 P fell in between untreated and heat-treated 15 P in terms of the proportion of each type of starch. There were no statistically significant differences in digestible starch content of 15 P across treatment types (*p* > 0.05). Overall, any pressure and heat-induced changes to the starch granule that may have occurred during the two treatments did not significantly affect starch digestibility during dynamic digestion. Supplementary Table 2c shows the glucose assay raw data.

## Discussion

Both HPP and heat treatment of proteins induce dissociation of subunits, denaturation, and rearrangement of secondary and tertiary structure, which can result in aggregation and formation of gel networks^1,3^. Structural modification and denaturation may expose previously inaccessible proteolytic cleavage sites in heat- and HPP-treated proteins, leading to greater digestibility; or extensive aggregation may occur, leading to lower digestibility^3^. Detailed information regarding structural changes induced in PPC by both HPP and heat treatment can be found in previous studies by our group^4,5^. Briefly, both treatments used in this study resulted in protein unfolding, followed by aggregation. The aggregates formed after the two treatments are likely to be different, due to the different nature of the forces responsible for aggregation, which are represented predominantly by disulfide bonds for heat-treated proteins, and predominantly non-covalent forces for HPP-treated proteins^4,5^. Since the latter forces are weaker, aggregates formed after HPP treatment are likely easier to break down during the digestion process, and thus become more accessible to proteolysis.

All assays conducted in this study indicated that HPP-treated PPC was more accessible for proteolysis during static gastric digestion than untreated and heat-treated PPC, at both protein concentrations. A similar increase in initial proteolysis by HPP-treatment was found in the first 20 min of jejunal and total digestion of 5 P during dynamic digestion. Pressure-induced denaturation in the pea proteins may have opened the protein structure in a way that enhanced substrate accessibility during initial enzymatic hydrolysis^17^. Increased initial proteolysis by HPP is interesting and may require further investigation, as rapid proteolysis has been linked to improved postprandial protein gain in elderly people^31^. However, both the static and dynamic digestion data showed that HPP and heat-induced structural changes in pea proteins only affected their digestibility during initial in vitro digestion, but not overall protein digestibility. Therefore, it is likely that structural changes induced by HPP or heat affected substrate accessibility for the initial proteolysis, but as proteins underwent more cleavage over time these structural differences did not affect overall protein digestibility by the completion of in vitro digestion.

Data also revealed differences in digestibility between HPP-treated and heat-treated PPC. Both processes can induce protein denaturation, structural modification by the formation of new intermolecular interactions, aggregation, and network formation^3,5,32^, all of which are likely to alter protein accessibility for proteolytic cleavage during digestion. The higher degree of proteolysis in the HPP-treated PPC compared to heat-treated PPC suggests that HPP treatment did not cause as extensive aggregation as heat treatment. Surface hydrophobicity in pressure and heat-treated proteins has been shown to increase during denaturation to a point and then start to decrease as exposed hydrophobic residues interact to form aggregates and disulfide bonds form^33–35^. Pepsin preferentially cleaves at hydrophobic residues^36^, so it is possible that more hydrophobic residues were accessible in the HPP-treated PPC compared to both untreated and heat-treated PPC during static gastric digestion. These differences can also explain the varied protein and peptide compositions of the digesta from the different treatments, observed in the SDS-PAGE analyses (Fig. 1a, b). We hypothesize that the predominantly weak physical bonds holding together the pressure-formed gels likely allowed a higher rate of gel dissolution compared to the heat-induced gels, which are held together primarily by covalent bonds.

Protein concentration impacted the way the two treatments affected digestibility. Previous work has shown that thermal treatment of pulses at low protein concentrations may increase protein digestibility, due to increased substrate accessibility for digestive proteases upon denaturation^37–39^. However, at higher concentrations, thermal gelation of soy protein isolate has been shown to lower the rate of gastric proteolysis^25^. Protein concentration impacts protein aggregate size and formation, due to increased availability of particular protein-protein interactions and crowding effects^40^, thus affecting proteolytic accessibility. Therefore, it is likely that increased protein–protein interactions and gel network formation in the high protein concentration (15 P) HPP- and heat-treated PPC somewhat decreased the susceptibility of proteins for enzymatic attack compared to the lower protein concentration samples. This may explain why HPP lowered initial ileal digestibility in 15 P and increased initial ileal digestibility in 5 P during dynamic digestion. Nevertheless, the effect of protein concentration on digestibility was neither consistent nor substantial.

The varying frameworks of the static and dynamic in vitro digestion systems likely contributed to incongruities in digestibility data between them, since sampling occurred at different digestion phases in the two systems. For the static model, sampling was not performed at multiple time points because removing a sample for analysis from the relatively small volume of digestion mixture would have altered the enzyme:substrate ratios, leading to inaccuracies in digestibility calculations. By comparison, the dynamic digestion model uses far larger digestion mixture volumes and is specifically equipped for sampling over time. The dynamic model, however, is not mechanically equipped for gastric or duodenal sampling, therefore digesta was only sampled from the latter two small intestine compartments, the jejunum and ileum. The two models also used different enzyme:substrate ratios and enzyme activity and had considerable differences in the shear forces affecting digesta movement. For these reasons, direct comparisons of the data obtained with the two models cannot be made. What is most relevant though are the trends in digestion observed with both models, which confirm the effects of the two treatments on PPC digestibility.

Another factor that can affect the digestibility of pea proteins is the presence of trypsin inhibitors, primarily of the Bowman–Birk family^41^; these compounds inactivate trypsin via formation of an enzyme-inhibitor noncovalent complex^42^ and thus lower protein digestibility. HPP did not inactivate trypsin inhibitors, and therefore any HPP-driven changes in digestibility throughout the in vitro digestions were likely due to structural changes. Other studies also reported that HPP treatments could not inactivate trypsin inhibitors in soy milk at 500 and 800 MPa for 2 min^43^. However, HPP induced partial inactivation in split peas and white beans at 600 MPa for 30–60 min at 20 °C^19^.

On the other hand, heat treatment lowered trypsin inhibitor activity by nearly 70% in 5 P (Fig. 5), which is consistent with previous studies that have shown that thermal treatment of pulses can inactivate the trypsin inhibitors^38,44,45^. Despite this, protein digestibility of heat-treated PPC was either lower than or comparable to untreated PPC, which suggests that aggregation post heat treatment was the predominant factor for digestibility.

As a note, peas have considerably lower trypsin inhibitor content compared to soy^46^, so trypsin inhibitor activity likely plays a smaller role in overall protein digestibility in peas as compared to soy.

Since starch is present in low concentrations in PPC, the effect of HPP on the digestibility of starch in PPC was evaluated in this study as well. Starch, including starch from pulses, has low susceptibility for enzymatic hydrolysis in its native state^47^ due to highly ordered amylopectin in the semicrystalline granules^48^. Pressure-treated starch retains a greater degree of granule structure than heat-treated starch^5,21^. Previous studies demonstrated an increase in starch digestibility after thermal processing^47,49^ and varied effects of pressure on starch digestibility^50–56^.

In the current study, treatment type did not have a significant effect on starch digestibility in 15 P, after both the static and dynamic digestion. At the low starch concentrations naturally present in PPC, pressure and heat-driven changes to the starch granule did not significantly affect starch hydrolysis to glucose. Even though there were no statistical differences in starch digestibility between treatments, the overall trends for dynamic starch digestion, with digestibility of heat-treated > HPP-treated > untreated (Fig. 6a–c), align with previous reports on starch digestibility^47,54–56^. One can speculate that the low concentration of starch in 15 P resulted in a high α-amylase:starch ratio such that any treatment-driven structural changes were outweighed by the high enzyme concentration. Most studies in which HPP treatment affected starch digestibility had far greater starch content in their samples compared to this study^50–56^.

This study indicates that HPP-induced structural modification of PPC does not affect the overall in vitro digestibility of protein or starch but may enhance gastric proteolysis. HPP can be used to create desirable structural and textural changes in proteins, which can facilitate the development of new food products. The fact that HPP does not negatively impact protein digestibility is important, and it brings further confirmation that this treatment does not have negative effects on food quality.

## Methods

### Materials and chemicals

PPC obtained by air classification (Pea Protein 55, AGT Food and Ingredients, Regina, SK, Canada) was used as the source of pea protein. The PPC powder composition was determined at Dairy One Laboratories (Ithaca, NY, USA), and consisted of: 54.5% (dry weight) protein, 4.3% (dry weight) starch, 2.8% (dry weight) fat, 6.7% (dry weight) ash, and 7.2% moisture. For the COST INFOGEST static in vitro digestion model, α-amylase from human saliva (A1031, Type XIII-A, 300–1500 U/mg), L-α-phosphatidylcholine from egg yolk (P3556, type XVI-E), pepsin A from porcine gastric mucosa (P7000, ≥250 units/mg), pancreatin from porcine pancreas (8× USP, P7545), porcine bile extract (B8631), and Pefabloc^®^ SC (76307) were obtained from MilliporeSigma (Burlington, MA, USA). For the TIM-1 in vitro gastro-intestinal dynamic model, type II-A α-amylase from *Bacillus* species (A6380, 1333 units/mg), pepsin A from porcine gastric mucosa (P7012, ≥2500 units/mg), lipase from porcine pancreas (L3126, Type II, 100–500 units/mg), and pancreatin from porcine pancreas (4× USP, P1750 and 8× USP, P7545) were obtained from MilliporeSigma (Burlington, MA, USA). Fresh pig bile was obtained from TNO Zeist (Netherlands). *o*-phthaldialdehyde (P1378), l-glutamine (PHR1125), Nα-Benzoyl-dl-arginine 4-nitroanilide hydrochloride (B4875), and trypsin from porcine pancreas (T4799, 1000–2000 units/mg) for the trypsin inhibitor activity assay were obtained from MilliporeSigma (Burlington, MA, USA). All chemicals were of analytical grade quality.

### Pea protein concentrate sample preparation

PPC samples with 5% and 15% (w/w) pea protein (5 P and 15 P, respectively) were prepared by stirring PPC in MilliQ water in the appropriate proportions to obtain the desired protein concentration, calculated based on the PPC composition provided above. This was followed by high shear mixing (18,000 rpm for 7.5 min) with an UltraTurrax Model T25 fitted with an S25N-18G dispersion tool (IKA Works Inc., Wilmington, NC, USA). 15 P samples contained 1.19% (w/w) starch. 5 P samples contained 0.40% (w/w) starch, which was deemed too low for starch digestibility analysis. 5 P and 15 P were vacuum-sealed (VP210 Chamber Vacuum Sealer, VacMaster, Overland Park, KS, USA) in 3 mm barrier vacuum pouches (cast nylon and polyethylene copolymer, containing vinyl acetate, Associated Bag, Milwaukee, WI, USA) and stored for ~24 h at 4 °C prior to HPP or heat treatments.

### HPP treatment

Samples of 5 P and 15 P were subjected to HPP treatment at 600 MPa and 5 °C for a holding time of 4 min, in a 55 L HPP unit (Hiperbaric, Spain), according to Sim and Moraru^4^. At the start of each HPP treatment, the temperature of the pressurizing medium (filtered water) was 5 °C. HPP-treated PPC was stored at 4 °C prior to digestion. Samples were digested within ~24 h of treatment.

### Heat treatment

Both 5 P and 15 P were heat-treated at 95 °C for 15 min via immersion in a water bath, immediately followed by cooling in an ice bath for 15 min, according to Sim et al.^5^, as these conditions were deemed sufficient for pea protein denaturation and gel structure formation^5,57,58^. Heat-treated PPC was stored at 4 °C prior to digestion. Samples were digested within ~24 h of treatment.

### COST INFOGEST static in vitro digestions

**Fig. 8:**
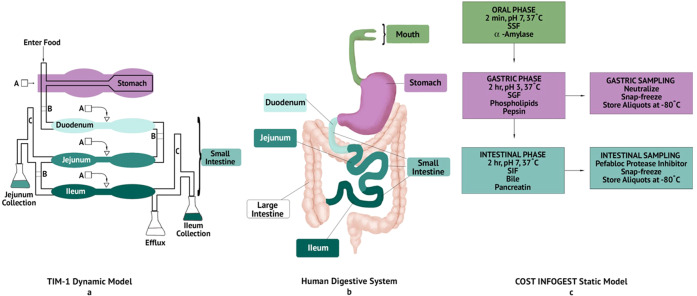
Comparison of the human GI tract to the dynamic and static in vitro digestion models. **a** The TNO gastrointestinal model 1 (TIM-1) dynamic in vitro digestion model (TNO Triskelion, Zeist, The Netherlands) contains four major compartments: the stomach, and the duodenum, jejunum, and ileum of the small intestine. Food enters the system and is exposed to simulated digestive secretions (**a**) as it flows through the system. Peristaltic valves (**b**) connect the major compartments and allow for the pumping of digesta throughout the system. Digestion products in the jejunum and ileum are filtered by dialysis through a hollow fiber system (**c**) for collection^60^. **b** A generalized overview of the human gastrointestinal tract. **c** Overview of the COST FA1005 Action INFOGEST standardized in vitro static digestion model. Each phase consisted of simulated digestive fluids (simulated salivary fluid, SSF; simulated gastric fluid, SGF; simulated intestinal fluid, SIF) and the major digestive enzymes at physiological activity levels^59^. Figure designed by Patty Rybolt of Patty Rybolt Designs; inspiration for panel (**a**) drawn from Minekus^60^.

Two in vitro digestion models were used to digest 5 P and 15 P: one static and one dynamic (Fig. 8). The untreated, HPP-treated, and heat-treated 5 P and 15 P underwent static in vitro digestion according to the COST FA1005 Action INFOGEST standardized protocol^59^ with minor modifications. Each unique lot of enzymes was assayed for activity level to ensure uniform enzyme activity across digestions. The activity levels of α-amylase and trypsin in pancreatin were determined as detailed in the supplementary materials of Minekus et al.^59^. Pepsin activity was measured according to the MilliporeSigma enzymatic assay of pepsin (3.4.23.1, MilliporeSigma, Burlington, MA, USA), with pepsin solution preparation as specified by the supplementary materials of Minekus et al.^59^. The concentration of bile acids in porcine bile extract was determined using a commercial bile acid assay kit (Bile Acid Assay Kit, MilliporeSigma, Burlington, MA, USA). Simulated salivary fluid (SSF), simulated gastric fluid (SGF), and simulated intestinal fluid (SIF) were all prepared according to Minekus et al.^59^. Digestions were maintained at 37 °C by the placement of the digestion beaker in an oil bath atop of a hot plate equipped with a temperature probe (VWR^®^ Professional Hotplate Stirrers, 97042-714, VWR, Radnor, PA, USA). To ensure congruous stir conditions, a mild stir at 390 rpm was maintained using the same hotplate, stir bar, and beaker for each digestion. The pH of the digestion mixture was monitored over time with a real-time data-logging pH sensor (Wireless pH sensor, PS 3204, Pasco Scientific, Roseville, CA, USA).

Each digestion replicate occurred as a pair of two separate digestions for gastric and intestinal sampling to ensure accurate analysis of digesta contents. The first consisted of the entire digestion protocol with digesta sampling at the end of the intestinal phase only (SI). Therefore, SI was the cumulative digesta after the intestinal phase of static in vitro digestion. The second digestion was terminated at the gastric phase, followed by a sampling of the cumulative gastric digesta (SG). See Table 1 for all symbol descriptions.

The digestion protocol consisted of three phases: oral, gastric, and intestinal. In the oral phase, 5 g of 5 P or 15 P (or distilled water blank) is first stirred with SSF for 1 min. For 15 P and blank digestions, α-amylase (75 units/mL digesta) and CaCl_2_ were added and stirred for exactly 2 min, followed by the start of the gastric phase. For 5 P digestions, the same protocol was followed except SSF was substituted for α-amylase as starch digestion was not analyzed. In the gastric phase, SGF, l-α-phosphatidylcholine, pepsin (2000 units/mL digesta), and CaCl_2_ were added to the digesta and the pH was maintained at 3. The substrate was exposed to pepsin for exactly 2 h, followed by either the start of the intestinal phase of gastric sampling. Gastric sampling entailed neutralization of the digested material to halt pepsin activity, snap-freezing with liquid nitrogen, and subsequent storage at −80 °C. In the intestinal phase, SIF, pancreatin (100 units/mL digesta based on trypsin), bile, and CaCl_2_ were added to the digesta and the pH was maintained at 7. The substrate was exposed to pancreatin for exactly 2 h, followed by intestinal sampling, where Pefabloc^®^ SC was added to inhibit serine protease activity, followed by snap-freezing with liquid nitrogen, and storage at −80 °C.

### TIM-1 gastro-intestinal dynamic in vitro digestion

The untreated, HPP-treated, and heat-treated 5 P and 15 P underwent dynamic in vitro digestion in the TIM-1 system (TNO, The Netherlands) at Rutgers University (New Brunswick, NJ, USA). Details of the system’s components, simulated digestive solutions, and enzyme solutions were outlined in previous works^60–62^. The TIM-1 system was composed of four successive glass compartments (stomach, duodenum, jejunum, and ileum) with flexible inner silicone membranes (Fig. 8). Digesta was mixed by mechanical imitation of peristaltic motion. Water at 37 °C was pumped into the space between the glass jacket and inner membrane to maintain human physiological temperature. One-hundred gram of 5 P or 15 P (or distilled water blank) feed was lightly stirred by hand with an electrolyte solution containing 11.5 mg α-amylase (1333 units/mg) and transferred to the stomach compartment. The digesta was then transported through the successive compartments via peristaltic valve pumps during the 240 min digestion. Gastric, duodenal, jejunal, and ileal secretions containing electrolyte, enzyme, and bile solutions were injected into the appropriate compartments by computer-controlled pumps. The gastric enzyme solution contained 0.05 g of lipase from the porcine pancreas (100–500 units/mg) and 0.04 g pepsin from the porcine gastric mucosa (≥2500 units/mg). The pancreatin solution contained 17.5 g pancreatin when 4× USP pancreatin from porcine pancreas was used in the first two replicates of 15 P digestions, and 9.15 g pancreatin when 8× USP pancreatin from porcine pancreas was used in the third 15 P replicate and all 5 P replicates. Both the 4× USP and 8× USP pancreatin powders were assayed for trypsin activity such that the same quantity of activity units was added for all digestions. Gastric and intestinal pH values were monitored and adjusted continually via the addition of HCl or NaHCO_3_. Gastric emptying, intestinal transit times, pH values, and secretion fluids were regulated by a computer protocol. Digesta was removed from the jejunum and ileum by dialysis through 10 kDa-cutoff hollow fiber membranes (0.05 μm pore, Spectrum Minikros, Repligen Corp., Boston, MA, USA) and collected as digesta pools for sampling (kept on ice during collection) at 20, 40, 60, 90, 120, 150, 180, 210, and 240 min after the start of digestion. Each digesta pool was weighed and frozen until further analyses.

Protein and starch content in the digesta pools collected at each time point for the dynamic digestions was summed for cumulative data over time. The cumulative contents of the jejunum, ileum, and total (jejunum + ileum) digesta from in vitro dynamic digestion at a given time point were labeled DCJ, DCI, and DCT, respectively, with subscripts indicating the time point of collection in min and treatment type. See Table 1 for all symbol descriptions.

### Static model digesta ultrafiltration

Prior to the BCA, OPA, and glucose assays, SG and SI were defrosted and underwent serial ultrafiltration in Amicon^®^ Ultra-4 centrifugal filter devices (MilliporeSigma, Burlington, MA, USA) with molecular weight cutoffs of 50 kDa, 30 kDa, and 10 kDa. Ultrafiltration occurred via centrifugation of digesta at 7500 × *g*, followed by a water rinse centrifugation step for each molecular weight cutoff centrifugal filter device. Ultimately, the SG and SI filtrate consisted of molecules less than 10 kDa. Therefore, all analyses, with the exception of SDS-PAGE, for both the dynamic and static model were performed on filtrate less than 10 kDa.

### Digestibility analyses

Protein assays were performed on the day of defrosting for the dynamic samples, and the day of defrosting and ultrafiltration for the static samples to avoid protein degradation from successive freeze–thaw cycles. Starch digestibility analyses were only conducted on 15 P digesta as the concentration of starch in the 5 P samples was low. Due to a large number of dynamic model samples from the various collection points, only the BCA and glucose assays were carried out for the dynamic model digesta. SDS-PAGE and the BCA, OPA, and glucose assays were performed on the static model digesta.

### SDS-PAGE analyses of digesta

Gel electrophoresis was performed at the Cornell University Institute of Biotechnology Proteomics and Metabolomics Facility (Ithaca, NY, USA) for unfiltered 5 P and 15 P static model digesta. Equal volumes of unfiltered SG, equal volumes of unfiltered SI, and untreated undigested PPC were loaded onto NuPAGE 12% bis-tris pre-cast polyacrylamide 1 mm gels (NP0343, Invitrogen^™^ by Thermo Fisher Scientific, Carlsbad, CA, USA). Proteins and peptides were separated by one-dimensional SDS-PAGE with NuPAGE 2-(n-morpholino) ethanesulfonic acid (MES) SDS running buffer (NP0002, Thermo Fisher Scientific, Waltham, MA, USA). Prior to loading, the untreated undigested PPC was reduced with DTT to separate protein subunits. PageRuler Plus pre-stained protein ladders were used for molecular weight markers (26619, Thermo Fisher Scientific, Waltham, MA, USA). After fixation, gels were stained with Colloidal Coomassie Blue (Colloidal Blue Staining kit, LC6025, Invitrogen^™^ by Thermo Fisher Scientific, Carlsbad, CA, USA). Gels were scanned with a Typhoon 9400 Variable Mode Imager (GE Healthcare Life Sciences, Marlborough, MA, USA) and analyzed via ImageQuant TL 8.1 software (GE Healthcare Life Sciences, Marlborough, MA, USA) to quantitate the total protein content in each well based on an *Escherichia coli* lysate standard curve. Relative protein concentrations (average protein concentration/average protein concentration of untreated) were calculated separately for SG and SI.

### Protein digestibility by BCA

The concentration of peptides (roughly tripeptides—10 kDa) released during in vitro digestion was determined using a bicinchoninic acid (BCA) protein assay kit (Pierce^TM^ BCA Assay, Thermo Fisher Scientific, Waltham, MA, USA). The BCA assay generally detects peptides that are at least three amino acids in length, with the exception of a few amino acids and dipeptides that can reduce Cu^2+^^63,64^. The microplate procedure was followed, according to the manufacturer’s instructions, for filtered 5 P and 15 P digesta of the dynamic and static models at each sampling point. Cu^2+^ was reduced upon formation of a Cu^2+^-tetradentate coordination complex with the peptide backbone, and the absorbance of a Cu^+^-bicinchoninic acid complex was measured at 562 nm^64^ (SpectraMax iD3, Molecular Devices LLC, San Jose, CA, USA). The concentration of peptides was determined from a bovine serum albumin (BSA) standard curve and the percent of initial protein input that makes up these peptides was calculated. The relative protein digestibility as determined by the BCA assay (RPD_BCA_) was calculated with respect to the average SI_Untreated_ percent digested protein for the static digestion (average % digested protein/average % digested protein of SI_Untreated_), and with respect to the average DCT_240,Untreated_ percent digested protein for the dynamic digestion (average % digested protein/average % digested protein of DCT_240,Untreated_). The Supplementary materials include further details regarding relative protein digestibility calculations.

A test was performed to ensure that the presence of reducing sugars would not interfere with the detection of protein by the BCA assay. Two standard curves were compared: one BSA only, and the other with the theoretical maximum concentration of glucose from PPC dissolved in the BSA solutions. The second curve was within the experimental error of the first. Therefore, the presence of reducing sugars did not affect the protein quantification by BCA.

### Protein digestibility by OPA

The concentration of small peptides (roughly < 6 kDa) and free amino acids in filtered 5 P and 15 P SG and SI was determined by the OPA assay according to Kopf-Bolanz et al.^65^, with some modifications. Specifically, 250 μL of 5 M perchloric acid and 750 μL of distilled water were added to 250 μL of filtered digested sample and incubated at 4 °C for 15 min. Following centrifugation at 10,000 × *g* for 5 min at 4 °C, 1.2 mL of OPA reagent (0.05 M borate, 10 g/L dodecyl sulfate, 0.8 g/L *o*-phthaldialdehyde, 5 g/L sodium 2-mercaptoethanesulfonate, and 5 g/L Triton X-100) was added to 40 μL of a combination of supernatant and distilled water in a ratio that ensured the absorbance remained within the linear range of detection. The reaction mixture was incubated in the dark at room temperature for 40 min. The absorbance at 335 nm was then measured (GENESYS^TM^ 20, Thermo Fisher Scientific, Waltham, MA, USA). The concentration of small peptides and free amino acids was calculated based on a glutamine standard curve and the percent of initial protein input that make up these peptides and free amino acids was calculated. The relative protein digestibility as determined by the OPA assay (RPD_OPA_) was calculated with respect to the average SI_Untreated_ percent digested protein (average % digested protein/average % digested protein of SI_Untreated_).

*O*-phthaldialdehyde and 2-mercaptoethanesulfonate form an adduct with free α-amino groups of free amino acids and peptides < 6 kDa^66^ with absorbance at 335 nm^67^. The specificity of this reaction for primary amines ensures negligible interference from other components in the food matrix. See Fig. 3e for a generalized representation of which protein hydrolysates are detected by the BCA and OPA assays.

### Starch digestibility

The concentration of glucose in the filtered digesta at each sampling point for the 15 P dynamic and static model digestions was determined using a glucose assay kit (Glucose (HK) Assay, MilliporeSigma, Burlington, MA, USA). This assay utilized hexokinase and glucose-6-phosphate dehydrogenase via substrate-specific reactions to minimize the interference of other components in the food matrix. The absorbance of NADH was measured at 340 nm (GENESYS^TM^ 20, Thermo Fisher Scientific, Waltham, MA, USA) to determine glucose concentrations in the digested samples, as NADH product is formed at a 1:1 stoichiometric ratio from glucose reactant. The percent of initial starch input that was hydrolyzed to glucose was calculated. From there, the relative starch digestibility (RSD) was calculated with respect to the average SI_Untreated_ percent digested starch for the static model (average % digested starch/average % digested starch of SI_Untreated_), and with respect to the average DCT_240,Untreated_ percent digested starch for the dynamic model (average % digested starch/average % digested starch of DCT_240,Untreated_). For the static model, starch digestibility was only measured for SI as starch digestion does not occur during the gastric phase.

For the dynamic model, starch content in 15 P was classified according to Englyst et al.^68^. Starch hydrolyzed to glucose in the first 20 min of digestion was deemed rapidly digestible starch. Starch hydrolyzed to glucose within 20–120 min of digestion was considered slowly digestible starch. Starch that was not hydrolyzed to glucose after 120 min of digestion was classified as resistant starch^68^.

### Trypsin inhibitor activity

Trypsin inhibitor activity was evaluated according to Kakade et al.^69^ with adaptations from Smith et al.^70^ and some other modifications. Trypsin inhibitors were extracted from the untreated, HPP-treated, and heat-treated 5 P by stirring 10 g of 5 P and 50 g of 0.01 M NaOH (pH adjusted to 9.5) for 3 h at room temperature. The solution was centrifuged at 12,500x*g* for 60 min and the supernatant was decanted.

The substrate solution was prepared by dissolving 0.1 g of Nα-Benzoyl-dl-arginine 4-nitroanilide hydrochloride (BAPNA) in 5 mL DMSO at 54 °C, followed by the addition of 0.05 M Tris-HCl (pH = 8.2) with 0.02 M CaCl_2_ up to 250 mL. The solution was stirred at 54 °C until clear, then kept at 37 °C prior to use. A 0.088 g/L trypsin solution was prepared in 1 mM HCl and stirred on ice prior to use. The concentration of the trypsin solution was determined such that the absorbance at 410 nm after the trypsin standard reaction did not exceed approximately 0.450, in order to minimize variability^71^.

The reaction involved mixing 2 mL of a combination of water and extracted trypsin inhibitor solution (at a ratio that ensured 40–60% trypsin inhibition), 2 mL of the trypsin solution, and 5 mL of the BAPNA substrate solution, followed by incubation for 10 min at 37 °C. The reaction was stopped by the addition of 1 mL of 30% acetic acid. The reaction mixture was filtered via 0.45 μm nylon filter and the absorbance of the filtrate was measured at 410 nm. The absorbance was compared to a trypsin standard, which was determined using the same procedure but with water substituted for the extracted trypsin inhibitors.

Trypsin inhibitor activity (mg trypsin inhibited/g protein) was calculated according to Smith et al.^70^, where the mass of trypsin inhibited was computed based on the principle that pure trypsin would give an absorbance of 0.0190 per μg trypsin^69^. Relative trypsin inhibitor activity was calculated with respect to the average untreated trypsin inhibitor activity (average trypsin inhibitor activity/average trypsin inhibitor activity of untreated).

### Statistical analysis

For the static in vitro digestion model, three independent pairs of digestions (where a pair was composed of separate gastric sampling and intestinal sampling digestions per sample) were made for each treatment (untreated, HPP-treated, heat-treated) of both 5 P and 15 P, and the blank (with 5 g of distilled water in replace of PPC). For the TIM-1 dynamic in vitro digestion model, three independent digestions were made for each treatment of both 5 P and 15 P, as well as the blank (with 100 g of distilled water in replace of PPC). Sample preparations and treatments were performed independently in triplicate. In order to minimize variance from sources other than treatment-type, digestions from both models were grouped in replicate sets, where each set consisted of an untreated, HPP-treated, and heat-treated digestion of either 5 P or 15 P. For each replicate set, PPC from one sample preparation was split into three different pouches that were either untreated, HPP-treated, or heat-treated. Digesta from each replicate set had similar storage durations and conditions, and they were defrosted and analyzed for digestibility on the same days. For the static model, each replicate set used the same α-amylase, pepsin, and Pefabloc^®^ SC solutions that were separated and frozen as aliquots; and digesta were defrosted and underwent centrifugation concurrently.

All digestibility analyses were performed independently on digesta from each independent set of replicates. Static model digestions were analyzed by BCA, OPA, and glucose assays in technical triplicate and SDS-PAGE in technical duplicate. Dynamic model digestions were analyzed by BCA and glucose assays in technical duplicate. Technical replicates were averaged to produce the data points for each independent digestion. Values reported are averages of the digestibility analyses from the three independent digestions ±SD (*n* = 3). Relative digestibility was calculated with respect to the average percent digested protein or starch values at the end of static or dynamic digestion for the untreated 5 P and 15 P.

Sample preparation, treatments, and assays were performed independently in triplicate for the trypsin inhibitor activity assay, and the trypsin inhibitor activity (mg trypsin inhibited/g protein) for each independent replicate was an average of three technical replicates. Values reported are averages of the three independent replicates ±SD (*n* = 3). Relative trypsin activity was calculated with respect to the average untreated 5 P trypsin inhibitor activity.

The raw data was analyzed using R statistical software 1.1.456 (R Core Team 2019, Vienna, Austria)^72^ with help from the statistical consulting unit at Cornell University. The static model data was fit to a linear model with a fixed effect for treatment and a random effect for replicate. The dynamic model data was fit to a linear model with fixed effects for treatment, time (as a categorical variable), and their interaction. Random effects were included for replicate and treatment within replicate to account for repeated measurements across time in the dynamic model. To fit linear model assumptions, the dynamic digestibility values were log-transformed prior to analysis. The trypsin inhibitor data was fit to a linear model with a fixed effect for treatment and random effects for replicate and technical replicates within replicates. The statistical models were fit using the lmerTest package in R^73^. Significance was determined with a two-tailed test at *α* = 0.05 using Tukey’s multiple comparison test implemented with the emmeans package in R^74^. The statistical model excluded the blank digestions as the comparison of interest was PPC digestibility between treatments.

### Reporting summary

Further information on research design is available in the Nature Research Reporting Summary linked to this article.

## Supplementary information

REPORTING SUMMARY

Supplementary discussion

## Data Availability

All data that supports the findings of this study are available in this published article and the associated Supplementary information.
