# Supplementary material for: Comparative effects of high pressure processing and heat treatment on in vitro digestibility of pea protein and starch
Source: NPJ Sci Food. 2022 Jan 12;6:2. doi: 10.1038/s41538-021-00116-0 (PMC8755827; doi:10.1038/s41538-021-00116-0)
Supplement: Supplementary file 2 — Supplementary discussion [file 41538_2021_116_MOESM2_ESM.pdf]

SUPPLEMENTARY DISCUSSION

Rationale for reporting relative digestibility

For digestions of the static and dynamic model, blank digestions (distilled water in replace of PPC) were performed to differentiate protein content from digested PPC and autolyzed enzymes. The major digestive enzymes used were larger than the 10 kDa molecular wight cutoff[30] of the filter that was used to filter digesta analyzed by the BCA and OPA assays. Therefore, peptides detected by the BCA and OPA assays in the blank digesta were primarily due to enzyme autolysis (supplementary table 1a and 1b, supplementary table 2a and 2b). Enzyme autolysis was apparent in the static model PPC digestions, as the percent digested protein data for the OPA assay was greater than 100% after the intestinal phase for both 5P and 15P digestions (supplementary table 1a and 1b). More protein mass from enzymes was added to the 5P and 15P digestions than protein mass from PPC, so even a small degree of enzyme autolysis could elevate the percent digested PPC values above 100%.

With the absence of substrate, blank digestions likely had a greater extent of enzyme autolysis and different kinetics for the release of these autolyzed peptides than the PPC digestions. In the static model SDS-PAGE, the weaker bands around 25 kDa and 20 kDa for SI_{Blank} compared to that of the PPC SI (Fig. 1a and 1b) suggests a greater extent of enzyme autolysis in the blank digestions. In addition, the higher percent digested protein in the blank versus 5P SI BCA data may suggest a greater degree of autolysis in the absence of substrate (supplementary table 1a). Therefore, the true degree of enzyme autolysis in the PPC digestions is unknown, but it was likely less than the blank digested protein values (Supplementary table 1a and 1b, supplementary table 2a and 2b). As a result, the protein digestibility data from the blank digestions was not subtracted from the protein digestibility data of the PPC digestions for either digestion model. Instead, relative

protein digestibility values were calculated. This is fitting for this study, as trends in relative protein digestibility as a function of treatment were of importance, rather than absolute values. El et al. also found notable enzyme autolysis during digestions of kefir and goat milk by the same static model[30].

Despite the lack of PPC, starch was detected in the blank digesta of the static and dynamic models, as glucose was present in the bile and enzyme powders used in the digestions (supplementary table 1b and 2c). A test was performed to determine the glucose content in the bile and enzyme powders used in the static model, and the results aligned with the glucose content of the static model blank digestions. For the dynamic model, the kinetics of this background glucose flow through the membrane was not likely congruous in blank digestions and 15P digestions. Therefore, the blank glucose quantities were not subtracted from the data of the dynamic model and relative starch digestibility was reported. For consistency, starch digestibility was calculated in the same manner for the static model.

Relative trypsin inhibitor activity data with respect to the average untreated trypsin inhibitor activity was reported for consistency (Supplementary table 3).

SUPPLEMENTARY TABLES

Supplementary Table 1. Static digestion raw data. Raw digestibility data for static model digestions of (a) 5P and (b) 15P. The BCA, OPA, and glucose assays were performed on each independent replicate of digestions in technical triplicate, where the percent protein digestibility for each independent digestion was an average of these technical triplicates. SDS-PAGE were performed in technical duplicate for each independent replicate of digestions. The SDS-PAGE protein concentration for each independent digestion was an average of these technical duplicates. Values represent the average of the three independent digestions ± SD. Superscript letters indicate statistical differences. SG and SI were analyzed by the statistical model separately. Blank digestions were not included in the statistical model as the model sought to analyze differences in digestion by treatment-type.

a

5P Static Digestion Raw Data

	SG				SI			
	**Untreated**	**HPP-treated**	**Heat-treated**	**Blank**	**Untreated**	**HPP-treated**	**Heat-treated**	**Blank**
**SDS-PAGE (μg/μL)**	2.61 ± 0.19[a]	1.53 ± 0.16[b]	2.34 ± 0.30[a]	0.19 ± 0.11	1.96 ± 0.13[a]	2.07 ± 0.09[a]	1.86 ± 0.17[a]	1.92 ± 0.29
**BCA (% Digested)**	23.86 ± 1.30[a]	26.97 ± 0.18[b]	23.41 ± 0.53[a]	4.48 ± 0.02	76.24 ± 0.95[a]	76.28 ± 1.24[a]	77.09 ± 0.72[a]	81.67 ± 6.60
**OPA (% Digested)**	19.63 ± 0.74[a]	23.87 ± 0.19[b]	19.12 ± 2.05[a]	not detected	246.80 ± 4.15[a]	249.96 ± 1.29[a]	246.07 ± 2.72[a]	181.26 ± 25.77

b

15P Static Digestion Raw Data

	SG				SI			
	**Untreated**	**HPP-treated**	**Heat-treated**	**Blank**	**Untreated**	**HPP-treated**	**Heat-treated**	**Blank**
**SDS-PAGE (μg/μL)**	8.86 ± 0.32[ab]	6.52 ± 1.25[a]	10.02 ± 1.42[b]	1.12 ± 0.28	3.15 ± 0.34[a]	2.99 ± 0.56[a]	2.70 ± 0.19[a]	2.08 ± 0.31
**BCA (% Digested)**	19.14 ± 0.64[a]	22.47 ± 0.71[b]	18.09 ± 0.53[c]	1.49 ± 0.01	38.07 ± 1.96[a]	37.36 ± 3.08[a]	37.34 ± 2.40[a]	27.22 ± 2.20
**OPA (% Digested)**	12.61 ± 1.86[a]	16.28 ± 0.44[b]	10.88 ± 0.19[a]	not detected	119.09 ± 14.19[ab]	122.28 ± 17.52[a]	115.69 ± 14.77[b]	60.42 ± 8.59
**Glucose (% Digested)**	-	-	-	-	133.59 ± 5.19[a]	134.91 ± 1.38[a]	132.67 ± 2.40[a]	56.71 ± 1.07

Supplementary Table 2. Dynamic digestion raw data. Raw digestibility data for the dynamic model (a) 5P protein, (b) 15P protein, and (c) 15P starch digestibility analyses. The BCA and glucose assays were performed on each independent replicate of digestions in technical duplicate, where the percent digestibility for each independent digestion was an average of these technical duplicates. Values represent the average of the three independent digestions ± SD. Blank digestions were not included in the statistical model as the model sought to analyze differences in digestion by treatment-type. Statistical analysis of effect of treatment type on digestibility was performed at each time point of the jejunum, ileum, and total (jejunum+ileum) digestion. Statistical comparisons between treatments in a given compartment at a given time point were not significant, unless designated with letter superscripts ($p<0.05$).

a

5P Dynamic Digestion Raw data: Protein Digestibility

	Cumulative Jejunum (% Digested Protein)				Cumulative Ileum (% Digested Protein)				Cumulative Total (% Digested Protein)			
**Time (min)**	Untreated	HPP-treated	Heat-treated	Blank	Untreated	HPP-treated	Heat-treated	Blank	Untreated	HPP-treated	Heat-treated	Blank
**20**	5.92 ± 0.70[a]	7.62 ± 0.39[b]	6.52 ± 0.91[ab]	7.54 ± 0.39	0.38 ± 0.06[a]	0.26 ± 0.16[ab]	0.20 ± 0.19[b]	0.54 ± 0.08	6.30 ± 0.75[a]	7.89 ± 0.34[b]	6.72 ± 1.01[ab]	8.09 ± 0.38
**40**	11.48 ± 0.90	14.19 ± 0.92	12.76 ± 1.60	13.66 ± 0.77	1.23 ± 0.53	0.93 ± 0.78	0.51 ± 0.44	1.31 ± 0.10	12.71 ± 1.11	15.12 ± 0.42	13.27 ± 1.79	14.97 ± 0.73
**60**	16.80 ± 1.45	20.40 ± 1.78	19.03 ± 2.27	18.96 ± 1.07	2.15 ± 1.23	1.66 ± 1.58	0.82 ± 0.65	1.99 ± 0.19	18.95 ± 1.49	22.06 ± 0.37	19.85 ± 2.60	20.95 ± 1.03
**90**	24.34 ± 2.47	28.56 ± 3.54	27.59 ± 2.66	25.21 ± 1.05	3.93 ± 1.99	3.58 ± 2.69	2.13 ± 0.91	3.06 ± 0.18	28.27 ± 1.99	32.14 ± 0.85	29.73 ± 3.33	28.27 ± 1.04
**120**	31.56 ± 3.10	35.52 ± 4.85	35.03 ± 2.88	29.69 ± 0.95	6.25 ± 2.21	6.17 ± 3.27	4.26 ± 0.92	4.61 ± 0.30	37.81 ± 2.56	41.69 ± 1.69	39.29 ± 3.64	34.30 ± 1.08
**150**	38.48 ± 3.56	42.25 ± 6.13	41.91 ± 2.79	33.65 ± 0.93	8.42 ± 2.44	8.50 ± 3.49	6.31 ± 0.98	5.93 ± 0.48	46.90 ± 3.16	50.75 ± 2.77	48.21 ± 3.66	39.59 ± 1.29
**180**	44.00 ± 3.71	47.92 ± 6.76	47.29 ± 2.65	37.06 ± 0.84	10.35 ± 2.62	10.59 ± 3.74	8.15 ± 1.05	7.04 ± 0.59	54.35 ± 3.49	58.52 ± 3.16	55.43 ± 3.61	44.10 ± 1.39
**210**	48.74 ± 4.03	53.03 ± 6.90	51.99 ± 2.62	40.37 ± 0.86	11.97 ± 2.82	12.44 ± 4.12	9.60 ± 1.10	8.03 ± 0.68	60.71 ± 4.09	65.47 ± 2.93	61.59 ± 3.64	48.40 ± 1.54
**240**	53.31 ± 4.45	57.81 ± 7.11	56.37 ± 2.62	44.02 ± 0.99	13.39 ± 2.98	13.93 ± 4.36	10.90 ± 1.12	9.03 ± 0.85	66.70 ± 4.74	71.75 ± 2.92	67.27 ± 3.64	53.04 ± 1.82

b

15P Dynamic Digestion Raw data: Protein Digestibility

Time (min)	Cumulative Jejunum (% Digested Protein)				Cumulative Ileum (% Digested Protein)				Cumulative Total (% Digested Protein)			
	Untreated	HPP-treated	Heat-treated	Blank	Untreated	HPP-treated	Heat-treated	Blank	Untreated	HPP-treated	Heat-treated	Blank
**20**	2.44 ± 0.12	2.43 ± 0.37	2.19 ± 0.09	2.51 ± 0.13	0.18 ± 0.04[a]	0.15 ± 0.03[a]	0.11 ± 0.04[b]	0.18 ± 0.03	2.62 ± 0.09	2.59 ± 0.39	2.30 ± 0.06	2.70 ± 0.13
**40**	4.91 ± 0.19	4.79 ± 0.69	4.46 ± 0.00	4.55 ± 0.26	0.50 ± 0.10[a]	0.31 ± 0.06[b]	0.33 ± 0.10[b]	0.44 ± 0.03	5.41 ± 0.10	5.10 ± 0.65	4.79 ± 0.10	4.99 ± 0.24
**60**	7.79 ± 0.36	7.80 ± 0.50	7.17 ± 0.21	6.32 ± 0.36	0.77 ± 0.11[a]	0.56 ± 0.07[b]	0.55 ± 0.14[b]	0.66 ± 0.06	8.56 ± 0.25	8.35 ± 0.43	7.72 ± 0.35	6.98 ± 0.34
**90**	13.49 ± 0.69	12.88 ± 0.99	12.18 ± 0.59	8.40 ± 0.35	1.66 ± 0.11	1.44 ± 0.07	1.44 ± 0.14	1.02 ± 0.06	15.15 ± 0.77	14.32 ± 0.95	13.61 ± 0.67	9.42 ± 0.35
**120**	19.94 ± 1.26	18.86 ± 1.21	17.80 ± 0.85	9.90 ± 0.32	3.41 ± 0.32	3.18 ±0.21	3.19 ± 0.18	1.54 ± 0.10	23.35 ± 1.57	22.04 ± 1.16	20.99 ± 0.80	11.43 ± 0.36
**150**	26.32 ± 1.66	25.14 ± 1.23	23.46 ± 0.92	11.22 ± 0.31	5.15 ± 0.54	4.95 ± 0.37	4.87 ± 0.27	1.98 ± 0.16	31.47 ± 2.17	30.08 ± 1.22	28.33 ± 0.68	13.20 ± 0.43
**180**	30.87 ± 2.15	29.62 ± 1.17	27.33 ± 0.94	12.35 ± 0.28	6.60 ± 0.75	6.47 ± 0.54	6.18 ± 0.45	2.35 ± 0.20	37.48 ± 2.85	36.09 ± 1.18	33.51 ± 0.60	14.70 ± 0.46
**210**	34.14 ± 2.58	32.84 ± 1.16	30.08 ± 0.95	13.46 ± 0.29	7.73 ± 0.84	7.68 ± 0.64	7.23 ± 0.49	2.68 ± 0.23	41.86 ± 3.31	40.52 ± 1.11	37.31 ± 0.58	16.13 ± 0.51
**240**	36.65 ± 2.91	35.32 ± 1.12	32.29 ± 0.94	14.67 ± 0.33	8.63 ± 0.89	8.64 ± 0.72	8.08 ± 0.57	3.01 ± 0.28	45.28 ± 3.68	43.96 ± 1.01	40.37 ± 0.58	17.68 ± 0.61

c

15P Dynamic Digestion Raw data: Starch Digestibility

	Cumulative Jejunum (% Digested Starch)				Cumulative Ileum (% Digested Starch)				Cumulative Total (% Digested Starch)			
**Time (min)**	Untreated	HPP-treated	Heat-treated	Blank	Untreated	HPP-treated	Heat-treated	Blank	Untreated	HPP-treated	Heat-treated	Blank
**20**	1.31 ± 0.61	1.36 ± 0.64	1.43 ± 0.24	1.22 ± 0.59	0.08 ± 0.05	0.07 ± 0.02	0.06 ± 0.03	0.07 ± 0.03	1.39 ± 0.65	1.43 ± 0.66	1.49 ± 0.25	1.29 ± 0.61
**40**	3.57 ± 1.06	3.68 ± 1.25	3.97 ± 0.16	2.19 ± 1.12	0.23 ± 0.11	0.21 ± 0.03	0.17 ± 0.06	0.17 ± 0.06	3.80 ± 1.18	3.89 ± 1.28	4.14 ± 0.21	2.36 ± 1.18
**60**	7.83 ± 1.22	8.89 ± 1.62	9.15 ± 0.94	2.98 ± 1.50	0.36 ± 0.16	0.37 ± 0.03	0.34 ± 0.08	0.26 ± 0.09	8.19 ± 1.37	9.25 ± 1.65	9.49 ± 0.95	3.24 ± 1.57
**90**	17.69 ± 1.03	19.56 ± 2.26	21.23 ± 3.40	3.95 ± 1.95	1.30 ± 0.34	1.69 ± 0.23	1.62 ± 0.14	0.44 ± 0.14	18.99 ± 1.07	21.24 ± 2.19	22.86 ± 3.51	4.39 ± 2.05
**120**	28.46 ± 2.53	32.43 ± 3.36	34.22 ± 5.78	4.63 ± 2.13	3.79 ± 0.37	4.86 ± 0.60	4.91 ± 1.11	0.62 ± 0.17	32.25 ± 2.41	37.28 ± 3.44	39.14 ± 6.80	5.26 ± 2.28
**150**	38.67 ± 4.21	44.33 ± 4.74	46.35 ± 8.50	5.21 ± 2.27	6.16 ± 0.24	8.09 ± 1.12	7.98 ± 1.70	0.75 ± 0.22	44.83 ± 4.02	52.42 ± 5.11	54.33 ± 10.03	5.96 ± 2.49
**180**	46.13 ± 4.93	53.54 ± 5.79	55.86 ± 10.15	5.78 ± 2.35	7.89 ± 0.46	10.72 ± 1.51	10.26 ± 1.71	0.82 ± 0.21	54.02 ± 4.49	64.26 ± 6.60	66.12 ± 11.62	6.60 ± 2.56
**210**	51.28 ± 5.64	59.87 ± 6.44	62.50 ± 10.32	6.21 ± 2.31	9.18 ± 0.45	12.63 ± 1.73	11.94 ± 1.83	0.88 ± 0.24	60.47 ± 5.34	72.50 ± 7.42	74.44 ± 11.89	7.09 ± 2.52
**240**	54.69 ± 6.30	64.13 ± 6.85	66.87 ± 9.45	6.57 ± 2.12	10.18 ± 0.40	13.94 ± 1.87	13.05 ± 1.63	0.95 ± 0.25	64.87 ± 6.42	78.08 ± 7.95	79.92 ± 10.75	7.51 ± 2.32

Supplementary Table 3. Trypsin inhibitor activity raw data.

	**Untreated**	**HPP-treated**	**Heat-treated**
**Trypsin Inhibitor Activity (mg trypsin inhibited/ g protein)**	7.37 ± 0.35[a]	7.44 ± 0.35[a]	2.32 ± 0.19[b]
